# Using deep learning to predict temporomandibular joint disc perforation based on magnetic resonance imaging

**DOI:** 10.1038/s41598-021-86115-3

**Published:** 2021-03-23

**Authors:** Jae-Young Kim, Dongwook Kim, Kug Jin Jeon, Hwiyoung Kim, Jong-Ki Huh

**Affiliations:** 1 Department of Oral and Maxillofacial Surgery, Gangnam Severance Hospital, Yonsei University College of Dentistry, Seoul, Republic of Korea; 2 Department of Oral and Maxillofacial Surgery, Yonsei University College of Dentistry, Seoul, Republic of Korea; 3 Department of Oral and Maxillofacial Radiology, Yonsei University College of Dentistry, Seoul, Republic of Korea; 4 Department of Radiological Science, Yonsei University College of Medicine, Seoul, Republic of Korea; 5 Department of Oral and Maxillofacial Surgery, Gangnam Severance Hospital, Yonsei University College of Dentistry, 211 Eonju-ro, Gangnam-gu, Seoul, 06273 Republic of Korea

**Keywords:** Oral manifestations, Oral diseases

## Abstract

The goal of this study was to develop a deep learning-based algorithm to predict temporomandibular joint (TMJ) disc perforation based on the findings of magnetic resonance imaging (MRI) and to validate its performance through comparison with previously reported results. The study objects were obtained by reviewing medical records from January 2005 to June 2018. 299 joints from 289 patients were divided into perforated and non-perforated groups based on the existence of disc perforation confirmed during surgery. Experienced observers interpreted the TMJ MRI images to extract features. Data containing those features were applied to build and validate prediction models using random forest and multilayer perceptron (MLP) techniques, the latter using the Keras framework, a recent deep learning architecture. The area under the receiver operating characteristic (ROC) curve (AUC) was used to compare the performances of the models. MLP produced the best performance (AUC 0.940), followed by random forest (AUC 0.918) and disc shape alone (AUC 0.791). The MLP and random forest were also superior to previously reported results using MRI (AUC 0.808) and MRI-based nomogram (AUC 0.889). Implementing deep learning showed superior performance in predicting disc perforation in TMJ compared to conventional methods and previous reports.

## Introduction

Disc perforation occurs in the late stage of temporomandibular joint (TMJ) disease. It may affect treatment planning and can be useful in predicting the prognosis of the disease^1–3^. Magnetic resonance imaging (MRI) is considered the gold standard for examination of disc of TMJs^2,4^. However, results among several reports predicting disc perforation based on MRI vary and the diagnostic accuracy of MRI in detecting TMJ disc perforation is known to be poor^2,3,5–7^.

Artificial intelligence (AI) technology is beginning to affect our daily lives, the field of medicine not excepted. Indeed, the number of articles applying machine learning to medical research has been growing rapidly in recent years^8,9^. Several studies have been also conducted to evaluate the diagnosis and prognosis in the field of oral and maxillofacial surgery. Zhang et al. reported a model that predicts postoperative facial swelling after third molar extraction with 98% accuracy using an artificial neural network^10^. Kim et al. also applied machine learning technique to predict the occurrence of bisphosphonate related-osteonecrosis of the jaw^11^.

In addition, deep learning, a class of machine learning, is increasingly being applied in the field of diagnosis and prediction related to medical imaging, yielding impressive results^12^. Yang et al. reported favorable result for automated detection of cyst and tumor of the jaw in panoramic images^13^. Lee et al. reported that cephalometric images can be applied for differential diagnosis of orthognathic surgery and orthodontic treatment based on deep convolutional neural networks with 95.4 ~ 96.4% success rate^14^. More recently, there are many attempts for diagnosis of osteoarthritis of the temporomandibular joint based on cone-beam computed tomographic image using machine learning^15,16^.

Although deep learning based on medical imaging has had a monumental impact, processing a stack of MRI slices rather than a single image such as a fundus photograph requires considerable effort as well as significant computational resources in terms of memory and processing speed^17–19^. Moreover, despite the ability of deep learning to extract features directly from data, predictions made solely by machines are limited in terms of accuracy and reliability; moreover, they raise legal, ethical, and psychosocial issues^8,9,12^.

Thus in this study, experienced observers interpreted the TMJ MRI images to extract data on specific features for use in building machine learning-based prediction models. The goal of this study was to construct machine learning models to predict TMJ disc perforation based on experienced investigators’ MRI readings and the validate the performance of the models. Random forest and multilayer perceptron (MLP), a class of deep learning method, were used. There have been to our knowledge no published studies applying deep learning or machine learning to TMJ disc perforation.

## Results

A total of 299 temporomandibular joints from 289 patients involved in this study. The characteristics of the study objects are shown in Table 1. In univariate analysis, there was significant difference statistically in all parameters except fluid collection between non-perforated and perforated group. A multiple logistic regression analysis was performed with parameters showing significance in the univariate analysis (Table 2). The factors significant in multivariable analysis were increased age, disc shape (eyeglasses or amorphous), low signal intensity of bone marrow in MRI, joint space, and changes in the condyle and fossa, consistent with a previous study^5^. Female patients were approximately twofold more likely to have disc perforation than male patients. When disc shape was amorphous, the possibility of disc perforation was increased almost 45-fold compared to normal disc shape.

**Table 1:** Characteristics of study objects.

	Non perforated group	Perforated group	*p*-value
Number of joints	N = 168	N = 131	
Age (year)***			< 0.001
Median [IQR]	27.0 [22.0; 33.0]	32.0 [26.5; 44.5]	
Gender*			0.029
Male	30 (17.9%)	10 (8.4%)	
Female	138 (82.1%)	120 (91.6%)	
Shape of the disc***			< 0.001
Biconcave	30 (17.9%)	1 (0.8%)	
Folded	46 (27.4%)	7 (5.3%)	
Flattened	34 (20.2%)	17 (13.0%)	
Eyeglass-shaped	40 (23.8%)	35 (26.7%)	
Amorphous	18 (10.7%)	71 (54.2%)	
Signal intensity of bone marrow***			< 0.001
Normal	152 (90.5%)	87 (66.4%)	
Low	16 (9.5%)	44 (33.6%)	
**Fluid collection**			0.178
Grade 0	82 (48.8%)	54 (41.2%)	
Grade 1	58 (34.5%)	52 (39.7%)	
Grade 2	24 (14.3%)	18 (13.7%)	
Grade 3	4 (2.4%)	7 (5.3%)	
Disc displacement***			< 0.001
Normal + ADcR	51 (30.4%)	7 (5.3%)	
Early ADsR	56 (33.3%)	31 (23.7%)	
Late ADsR	61 (36.3%)	93 (71.0%)	
Joint space***			< 0.001
Normal	38 (22.6%)	7 (5.3%)	
Narrowing	125 (74.4%)	91 (69.5%)	
Bone to bone contact (close)	3 (1.8%)	12 (9.2%)	
Bone to bone contact (open)	2 (1.2%)	21 (16.0%)	
Changes of condyle and fossa***			< 0.001
2 or less features	149 (88.7%)	72 (55.4%)	
2 or more features	19 (11.3%)	58 (44.6%)	

*IQR* Interquartile range, *ADcR* anterior displacement with reduction, *ADsR* anterior displacement without reduction.

*p < 0.05, **p < 0.01, ***p < 0.001.

**Table 2:** Univariate and multiple logistic regression analyses.

Variables	Univariate		Multiple	
	OR (95% CI)	*p*-value	OR (95% CI)	*p*-value
Age	1.06 (1.04–1.09)	0.000***	1.05 (1.02–1.09)	0.002**
**Sex**				
Male	1		1	
Female	2.37 (1.17–5.14)	0.021*	2.04 (0.76–5.83)	0.276
**Shape of the disc**				
Biconcave	1		1	
Folded	4.57 (0.76–87.59)	0.165	1.12 (0.13–24.74)	0.892
Flattened	15.00 (2.82–278.31)	0.011*	6.27 (0.98–123.82)	0.075
Eyeglasses-shaped	26.25 (5.19–479.44)	0.002**	11.72 (1.55–251.4)	0.040*
Amorphous	118.33 (22.97–2178.49)	0.000***	44.92 (5.84–975.71)	0.002**
**Signal intensity of bone marrow**				
Normal	1		1	
Low	4.80 (2.61–9.25)	0.000***	3.47 (1.48–8.56)	0.005**
**Fluid collection**				
G0	1			
G1	1.36 (0.82–2.27)	0.234		
G2	1.14 (0.56–2.29)	0.716		
G3	2.66 (0.76—10.55)	0.133		
Disc displacement				
Normal + ADcR	1			
Early ADsR	4.03 (1.71–10.69)	0.002**	1.45 (0.45–4.93)	0.367
Late ADsR	11.11 (5.01–28.28)	0.000***	0.94 (0.25–3.63)	0.898
**Joint space**				
Normal	1		1	
Narrowing	3.95 (1.79–10.02)	0.002**	3.21 (1.09–10.74)	0.066
Bone to bone contact (close)	21.71 (5.39–115.90)	0.000***	9.19 (1.51–70.15)	0.047*
Bone to bone contact (open)	57 (13.09–413.06)	0.000***	45.4 (6.53–497.03)	0.000***
**Changes of condyle and fossa**				
2 or less	1		1	
2 or more	6.23 (3.51–11.47)	0.000***	3.79 (1.75–8.58)	0.002**

*ADcR* anterior displacement with reduction, *ADsR* anterior displacement without reduction.

*p < 0.05, **p < 0.01, ***p < 0.001.

**Figure 1:**
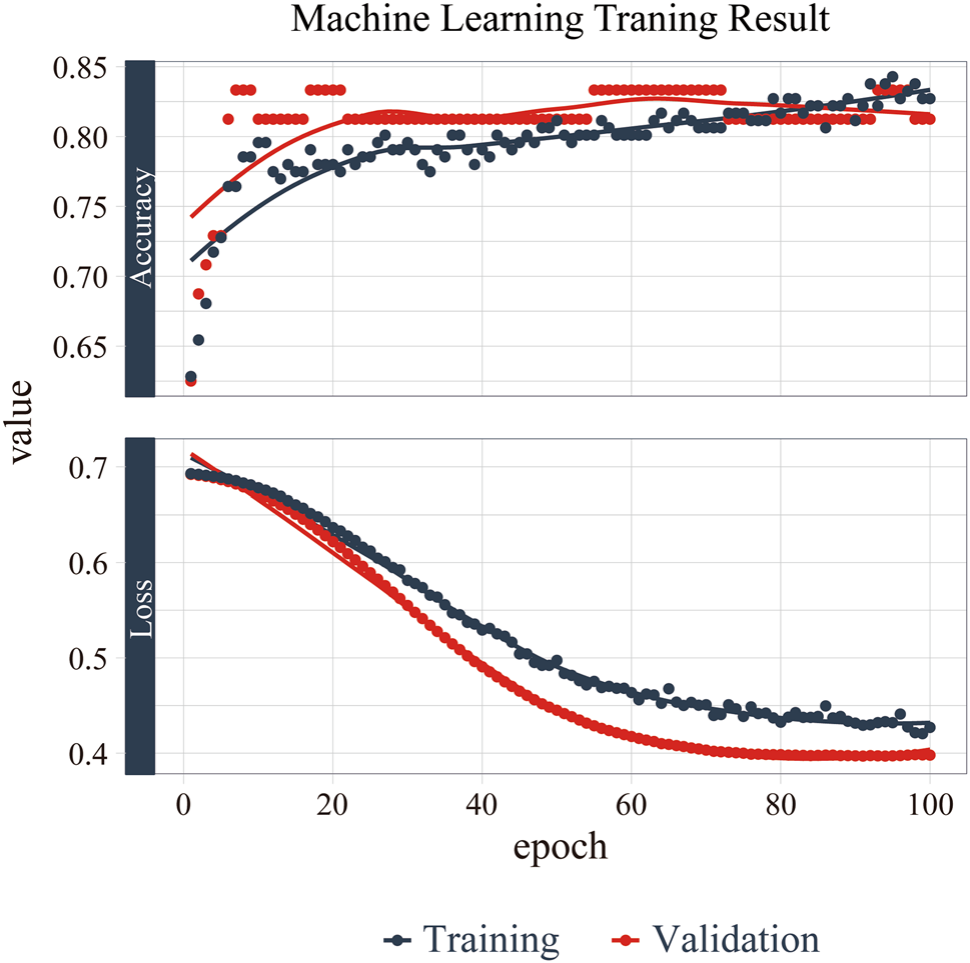
Training and validation curves. The training progress is shown by plotting the loss of each iteration. The accuracy of the validation set is also plotted together for every epoch.

**Figure 2:**
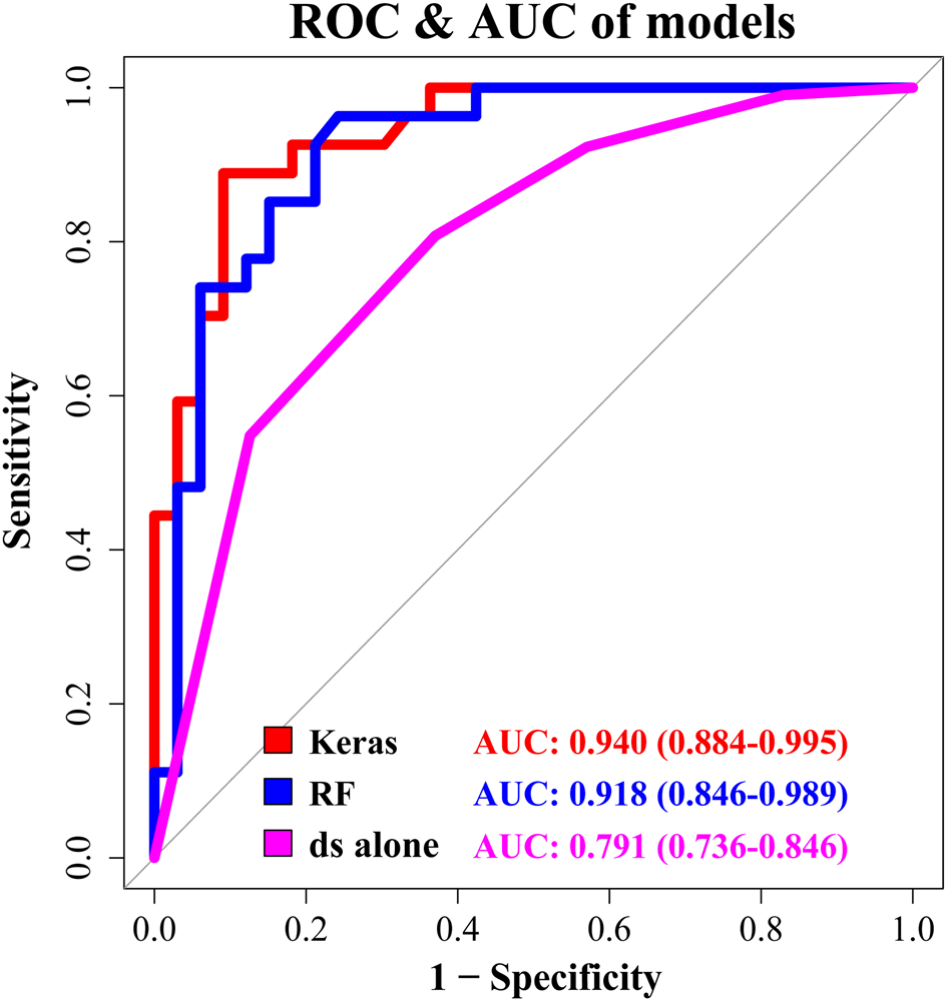
ROC and AUC of machine learning methods. MLP performed best (AUC 0.940), followed by random forest (AUC 0.918) and disc shape alone (AUC 0.630). *MLP* multilayer perceptron, *RF* random forest, *ds* shape of the disc.

The machine learning models were built based on the above results. The training progress is shown by plotting the loss of each iteration in Fig. 1. Statistically significant factors from the above analyses were considered when constructing the random forest and MLP models. MLP produced the highest performance (AUC 0.940), followed by random forest (AUC 0.918) and disc shape alone (AUC 0.791). (Fig. 2, Table 3). The AUC of MLP and random forest outperformed previous reports. (AUC 0.808^3^, AUC 0.889^5^).

**Table 3:** Statistical significance of the difference between the areas under ROC curves. DeLong’s test and Bootstrap test were used.

	Random forest (*p*-value)	Disc space alone (*p*-value)
MLP	0.344	< 0.01*
Random forest		< 0.01*

*MLP* multilayer perceptron.

**Figure 3:**
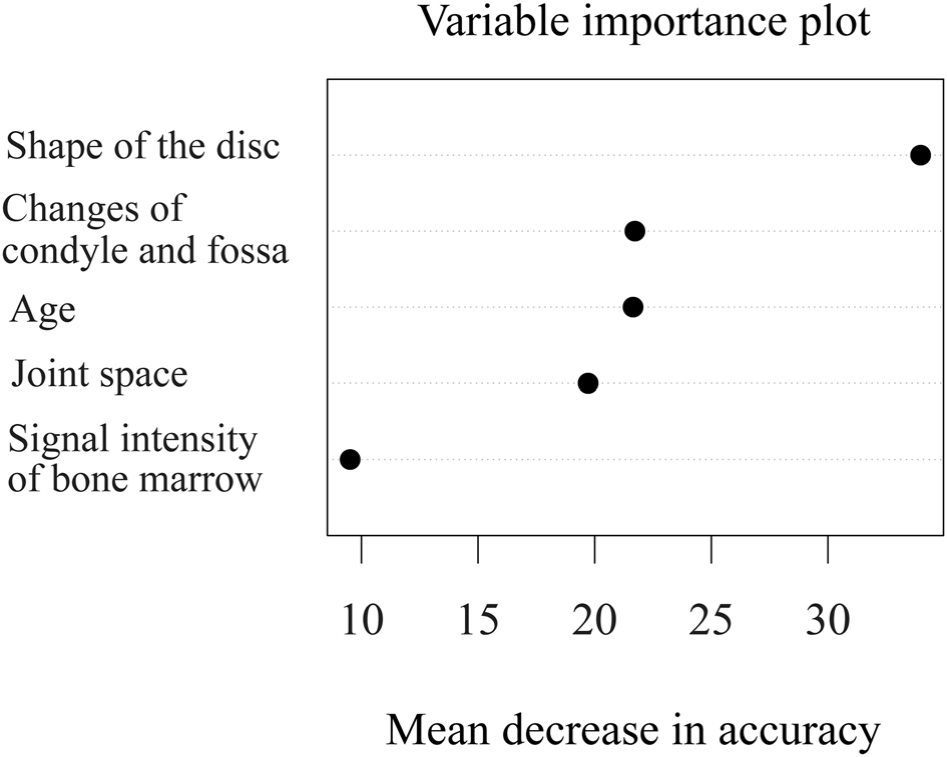
Plot showing importance of the variables in the random forest model. The importance is determined by the amount of reduction in uncertainty caused by arranging a variable in the tree structure. If a variable causes a large reduction in uncertainty, it occurs high up in the classification hierarchy and should thus be considered important.

The random forest model does yield the importance of the variables in each model, as shown in Fig. 3. According to the result of our model, shape of the disc appeared to have the most impact among the variables which were significant in multiple logistic analysis.

The sensitivity and specificity of each model at its optimal cutoff is also investigated. MLP model showed 85.2% of sensitivity and 84.8% of specificity. Random forest model showed 96.3% of sensitivity and 75.8% of specificity. They are summarized in Table 4.

**Table 4:** Sensitivity and specificity of each model at optimal cutoff point.

Model	Sensitivity	Specificity	Cutoff point of probability
MLP	85.2%	84.8%	0.508
Random forest	96.3%	75.8%	0.196
Disc shape alone	80.8%	63.0%	0.307

Optimal cutoff was considered the point maximizing the sum of sensitivity and specificity.

*MLP* multilayer perceptron.

## Discussion

Presence of temporomandibular disc perforation does not necessarily mean the patient needs surgical intervention. However, identification of TMJ disc perforation may affect treatment planning since it can be seen in the late stage of TMJ arthrosis and the stage of internal derangement affects treatment planning^2^.

Although MRI is widely considered the gold standard in evaluating TMJ, clear criteria for diagnosing TMJ disc perforation have not yet been proposed^3,5,7^. The diagnostic value of MRI for identifying the presence of a perforated disc is reported to be limited, only a handful of reports having assessed it with ROC curves^2–4^.

Previously, Shen et al. reported an AUC of 0.808 (95% CI 0.77–0.85) by diagnosing TMJ perforation with MRI^3^. One thing to consider is that this study included 2524 joints, but only 207 joints were perforated. This imbalance between the numbers of cases and controls allows high diagnostic accuracy, exceeding 90%, despite the sensitivity being as low as 0%. In other words, if there are only 10 perforated joints among 100 joints, diagnostic accuracy of 90% is easily achieved by simply diagnosing every joint as non-perforated, while none of the perforated joints are diagnosed correctly. The study still has significance in that it is the first report assessing efficacy of MRI-based diagnosis of TMJ disc perforation based on ROC curve rather than only sensitivity and specificity. The ROC curve is an efficient method for assessing a diagnostic test as it visualizes all possible combinations of true positive rates and false positive rates.

A previous study which constructed a nomogram based on MRI findings reported the AUC to be 0.889 (95% CI 0.804–0.973)^5^. In this study, the best performing deep learning model showed an AUC of 0.940 (95% CI 0.884–0.995). This is the highest result reported, despite being validated with mutually exclusive datasets from the training set.

Deep learning is yielding quantum leaps in a wide range of technologies affecting our lives. Face recognition, voice-to-text, personal assistants, and natural language understanding have become commonplace, and self-driving cars are on the horizon. The field of medicine is no exception: as the number of healthcare startups using artificial intelligence is increasing, so are markets involving this technology rapidly emerging. Across the board, medical imagery will likely constitute a primary input for practical applications using AI in healthcare.

Classic data-driven approaches in radiology depend on features seen as important from a human radiologist’s point of view, such as density, heterogeneity of tumors, shape, etc^20^. Convolutional neural network (CNN), a class of deep learning, automatically discovers the best features for a given task without requiring human-mediated feature selection^12,20^. CNN reaches or sometimes exceeds human performance in specific tasks.

While this study implemented a deep learning approach to construct a TMJ disc perforation detection model, feature selection and assessment were done by doctors and not by computer. A large amount of data is required for a well-performing CNN, which automates the feature selection process. Machine learning will only get better over time as data sets increase in size and computing power grows^21^. Given the small amount of available data, deep learning with human-crafted features performs better^20^. Moreover, it does not require extensive computing power as well. Also, it has been noted that even minor changes to the input data, often invisible to human eye, can result in dramatically different classifications^20,22^. Human verification is thus still required.

The “black box” nature of AI-based diagnosis, meaning the inability to identify the reason for each decision, is another limitation^9^. Doctors will rarely follow the advice of a machine if they cannot see the reasoning underlying that advice, especially when the responsibility for the patient remains with the clinicians^9,23^. There are ongoing studies on this, some of them achieving a measure of interpretability. However, though not fully interpretable, the random forest model does yield the importance of the variables in each model, as shown in Fig. 3.

In this study, temporomandibular disc perforation was confirmed by surgeon during open TMJ surgery. In some cases, however, the perforation was not identified according to the size or location of disc perforation, which is a limitation of this study. It is thought that the most optimal condition is to interpret and analyze all images by AI technique, if disc perforation can be diagnosed more completely through image. Recently, Chauhari et al. reported that super-resolution magnetic resonance images were created by deep learning and the images can be used for diagnosis^24^. With the advancement of AI technology and the development of image technology, it is expected that image interpretation and prediction of AI alone in the future.

Machine learning approaches are increasingly finding application in the field of medicine and will benefit patients whose doctors have learned to implement them. The history of medicine is replete with cases in which new techniques have been adopted by a few with notable success and then become widespread. To aid readers wishing to try out this new technology, we are sharing the R code used in this study as an attachment (Supplementary Data).

## Methods

### Patients

443 patients received open TMJ surgery between January 2005 and January 2018 at the Department of Oral and Maxillofacial Surgery, Gangnam Severance Hospital, Yonsei University. The following criteria were excluded: diagnosed tumors such as osteochondroma and synovial chondromatosis; a congenital deformity such as hemifacial microsomia or hemifacial hyperplasia; the absence or inadequate quality of TMJ MRI; and prior total TMJ replacement or arthroplasty.

This retrospective study was approved by the Institutional Review Board of Gangnam Severance Hospital (No. #3-2018-0129) and complied with the tenets of the Declaration of Helsinki. Written or verbal informed consent was not obtained from any participants because the IRB of Gangnam Severance Hospital waived the need for individual informed consent, as this study had a non-interventional retrospective design and all data were analyzed anonymously.

Following this selection process, 299 joints from 289 patients remained. These were divided into two groups, perforation and non-perforation, based on the existence of disc perforation identified during surgery. The characteristics of the study objects are shown in Table 1.

### Statistical analyses

Comparison of parameters between groups in Table 1 were assessed using the chi square test, Fisher’s exact test, and the Cochran-Armitage trend test for categorical variables. The Mann–Whitney rank sum test was used for continuous variables. Statistical analyses were performed using the R programming language (R Core Team, Vienna, Austria, 2018).

### Magnetic resonance imaging

MRI was acquired on a 3.0-T Magnetom scanner (Achieva, Philips Medical, Best, The Netherlands) with 3-inch surface coils for the TMJs. For T1-weighted imaging, the following parameters were used: repetition time, 450 ms; echo time, 20 ms; slice thickness, 3 mm; field of view, 120 mm; and acquisition matrix size, 240 × 240. The parameters for T2-weighted imaging were as follows: repetition time, 2,900 ms; and echo time, 90 ms. Sagittal plane MRI was analyzed by two oral and maxillofacial surgeons and an oral and maxillofacial radiologist with reference to previous studies^3,5,7,25^. All observers were experienced in TMJ MRI interpretation. Images were interpreted at the same time and a final decision was made by consensus.

Disc shapes, bone marrow signal, relationship between the disc and condyle, joint space, and changes of condyle and fossa were investigated. More detailed information and figures of each parameter have been described in a previous report^5^. The data derived from these features were used to build a prediction model.

### Disc shapes

“Biconcave” refers to the normal disc structure and position. “Folded” describes discs with either a cap- or cup-shaped (∩ -or ∪ -shaped) configuration. A “flattened” disc has a loss of the voluminous configuration of the anterior band, posterior band, or both. A disc shortened antero-posteriorly resembles a pair of eyeglasses and was named as such. A deformed disc without a distinguishing configuration was classified as “amorphous.” A disc falling into more than 2 of the above categories was classified into the more deformed category.

### Signal intensity of the bone marrow

Signal intensity of the bone marrow was assessed based on T1-weighted images. When the signal intensity of the condyle was lower than that of the ramus or body of the mandible, it was considered a low bone marrow signal; otherwise it was considered normal.

### Fluid collection

Fluid collection was considered present if high signal intensity was observed within the joint spaces on at least two consecutive T2-weighted sagittal MRI. The amount of collected fluid was divided into 4 grades, from G0 to G3, where G0 refers to no fluid, G1 limited to the verge of the disc, G2 extended over the verge, and G3 when capsular expansion was observed.

### Joint space

The narrowing of joint space between the condyle and fossa was divided into 4 categories: normal, narrowing, bone-to-bone contact while mouth closed, and bone-to-bone contact on mouth opening.

### Changes of condyle and fossa

The presence of the following 5 features of mandibular condyle and 1 feature of articular fossa, 6 features in all, were investigated: osteophyte, erosion, sclerosis, flattening, and superiorly forming bony projection (spur) of mandibular condyle and signal changes of articular fossa^2,5,7^. The number of features were counted.

### Machine learning

Prior to formulation of machine learning models, the data set was randomly divided into two mutually exclusive sets, training (80%) and validation (20%)^26^. The training set was used to construct the prediction model and the validation set was used to validate the performance of each model. Area under the Receiver Operating Characteristic (ROC) curve (AUC) was used to compare the performance of the models with one another, and also with those in previous reports.

A concise description of each machine learning algorithm is provided below. All machine learning models were implemented using the Keras framework^27^ with the R programming language (R Core Team, Vienna, Austria, 2016)^26^.

### Random forest

Random forest is a tree-based machine learning algorithm which creates subsets of decision trees and combines weak outputs of the trees to yield highly accurate results by calculating the vote of each tree^28^. Each decision tree predicts the value of a target variable based on several input variables by repeated classification, also known as recursive partitioning^28,29^. The subsets are created by multiple iterations of random sampling, 500 times in this study. The R package randomForest was employed^28^.

### Multilayer perceptron (MLP)

Multilayer perceptron (MLP) is a class of artificial neural network (ANN) with multiple, or deep, layers of nodes. Each neuronal node connects with others in patterns similar to those in animal neurons and uses a non-linear activation function. This non-linear characteristic makes it possible to distinguish linearly inseparable data. The Keras framework^27^, a recent deep learning interface, was employed to construct an MLP model in this study.

**Figure 4:**
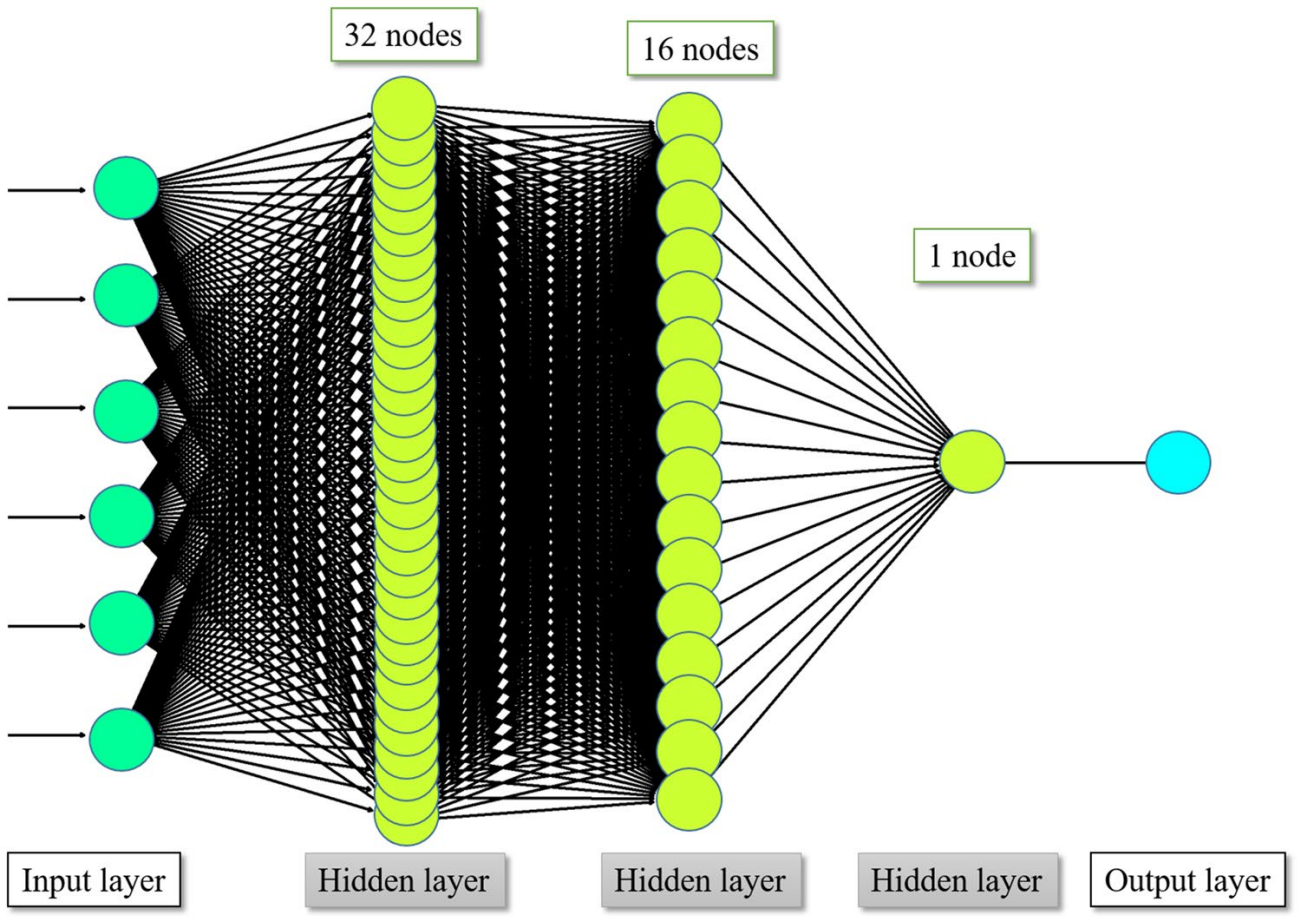
Architecture of multilayer perceptron (MLP). This deep neural network consists of an input layer, three fully connected hidden layers with nodes numbering 32, 16, and 1, and an output layer. When a series of training samples is presented to the network, a loss function measures the inaccuracy of the computed prediction. All parameters are then slightly updated in the direction that will minimize the error, a process called back-propagation.

The MLP model architecture used in this study, illustrated in Fig. 4, is composed of an input layer, three fully connected hidden layers and an output layer. The input layer refers to input data such as features extracted from TMJ MRI. The hidden layers are those where the input features are computed. The node in the output layer represents the computed prediction result^27^.

A neural network is trained by adjusting the weights and biases of each node. These parameters are repeatedly adjusted via an optimization algorithm called gradient descent^27^. Each time predictions are computed from a given data sample (forward propagation), the network performance is assessed through a loss function that measures the error of the prediction. Each network parameter is then adjusted in small increments in the direction that minimizes the loss, a process called back-propagation^27,30^. This MLP learning process is shown in Fig. 1.

## Conclusion

Implementing deep learning showed superior performance in predicting disc perforation in TMJ compared to conventional methods and previous reports.

## Supplementary Information

Supplementary Information.
