# Supplementary material for: Using deep learning to predict temporomandibular joint disc perforation based on magnetic resonance imaging
Source: Sci Rep. 2021 Mar 23;11:6680. doi: 10.1038/s41598-021-86115-3 (PMC7988137; doi:10.1038/s41598-021-86115-3)
Supplement: Supplementary file 1 — Supplementary Information. [file 41598_2021_86115_MOESM1_ESM.pdf]

Supplemental Materials

Using deep learning to predict temporomandibular joint disc perforation based on magnetic resonance imaging

Jae-Young Kim[1], Dong Wook Kim[2], Kug-Jin Jeon[3], Hwiyoung Kim[4], Jong-Ki Huh[5]

J.Y. Kim and D.W. Kim contributed equally as first authors to this work.

1Department of Oral and Maxillofacial Surgery, Gangnam Severance Hospital, Yonsei University College of Dentistry, Republic of Korea

2Department of Oral and Maxillofacial Surgery, Yonsei University College of Dentistry, Republic of Korea

3Department of Oral and Maxillofacial Radiology, Yonsei University College of Dentistry, Republic of Korea

4Department of Radiological Science, Yonsei University College of Medicine, Republic of Korea

5Department of Oral and Maxillofacial Surgery, Gangnam Severance Hospital, Yonsei University College of Dentistry, Republic of Korea

Correspondence

Jong-Ki Huh

Department of Oral and Maxillofacial Surgery, Gangnam Severance Hospital, Yonsei University College of Dentistry, 211 Eonju-ro, Gangnam-gu, Seoul 06273, Korea

Tel: +82-2-2019-4560

Fax: +82-2-3463-4052

E-mail: OMSHUH@yuhs.ac

Table of Contents

1. Loading the dataset 4
   1.1. Loading the raw data 4
   1.2. Checking the first 6 rows of the dataset 'tmj' 4
   1.3. Checking the structure of the dataset 'tmj' 4
   1.4. Changing column names 4
   1.5. Converting categorical variables into factors (currently integers) 4
2. Data Preprocessing 5
   2.1. Converting variables into numerical values for neural network 5
   2.2. Scale 5
   2.3. Partition of the data set into 80% training and 20% testing set 5
3. Random forest (RF) 5
   3.1. Load package "randomForest" 5
   3.2. Build model & predict 5
   3.3. Looking inside the RF model 5
   3.4. Importance of variables 6
   3.5. Importance plot 6
   3.6. Export into .eps format 7
4. Deep learning with keras 7
   4.1. Loading libraries 7
   4.2. trainging and test sets for keras 9
   4.3. Predictor variable for the training and testing set 9

4.4. Target Variable for training and testing set 9
4.5. Multi-Layer Perceptron 9
4.6. Compile 9
4.7. Fit the model to the training Data 10
4.8. Plotting the training / validation history of our Keras's Model 10
4.8.1. Export into .eps format 11
4.9. Performance 11
4.9.1. Predicted class 11
4.9.2. Predicted class probability 11
4.9.3. Format test data and prediction 11
4.9.4. Confusion table 12
4.9.5. Accuracy 12
4.9.6. AUC of ROC 12
5. Plotting ROC with AUC of all Machine Learning models + single predictors 12
5.1. Testing if the differences between models are statistically significant 14
6. Sensitivity, Specificity at Optimal cutpoints for each model 15
6.1. keras model 15
6.2. RF model 15
6.3. ds alone 16
7. Testing with imaginary data 16
7.1. Scale imaginary data to the scale of previous dataset 16
7.2. Predictor & Target variable for the imaginary data set 17

7.3. Predicted class ... 17

7.4. Predicted class probability ... 17

7.5. format test data and prediction ... 17

1. Loading the dataset

1.1. Loading the raw data

```
Sys.setlocale("LC_ALL", "English")

## [1] "LC_COLLATE=English_United States.1252;LC_CTYPE=English_United Stat
es.1252;LC_MONETARY=English_United States.1252;LC_NUMERIC=C;LC_TIME=Englis
h_United States.1252"

tmj <- read.csv("D:/Perforation_ML3.csv", sep=",", header=TRUE)
```

1.2. Checking the first 6 rows of the dataset 'tmj'

```
head(tmj)

##   Group Age Disc_Shape BMS JS Condyle_Fossa
## 1     0  24          3   1  2             0
## 2     1  53          5   1  2             0
## 3     0  21          1   1  2             0
## 4     0  71          1   1  1             0
## 5     0  23          4   1  2             0
## 6     1  64          5   1  3             0
```

1.3. Checking the structure of the dataset 'tmj'

```
str(tmj)

## 'data.frame':    299 obs. of  6 variables:
##  $ Group        : int  0 1 0 0 0 1 1 1 0 1 ...
##  $ Age          : int  24 53 21 71 23 64 54 70 58 43 ...
##  $ Disc_Shape   : int  3 5 1 1 4 5 5 5 5 5 ...
##  $ BMS          : int  1 1 1 1 1 1 2 1 1 1 ...
##  $ JS           : int  2 2 2 1 2 3 2 3 2 3 ...
##  $ Condyle_Fossa: int  0 0 0 0 0 0 0 0 1 1 ...
```

1.4. Changing column names

```
colnames(tmj)=c("Group","age","ds","bms","joint","bone")
```

1.5. Converting categorical variables into factors (currently integers)

```
tmj$ds <- as.factor(tmj$ds)
tmj$bms <- as.factor(tmj$bms)
tmj$joint <- as.factor(tmj$joint)
tmj$bone <- as.factor(tmj$bone)
str(tmj)
```

```
## 'data.frame':    299 obs. of  6 variables:
##  $ Group: int  0 1 0 0 0 1 1 1 0 1 ...
##  $ age  : int  24 53 21 71 23 64 54 70 58 43 ...
##  $ ds   : Factor w/ 5 levels "1","2","3","4",..: 3 5 1 1 4 5 5 5 5 5 ...
##  $ bms  : Factor w/ 2 levels "1","2": 1 1 1 1 1 1 2 1 1 1 ...
##  $ joint: Factor w/ 4 levels "1","2","3","4": 2 2 2 1 2 3 2 3 2 3 ...
##  $ bone : Factor w/ 2 levels "0","1": 1 1 1 1 1 1 1 1 2 2 ...
```

2. Data Preprocessing

2.1. Converting variables into numerical values for neural network

```
tmj$Group<-as.numeric(tmj$Group)
tmj$age<-as.numeric(tmj$age)
tmj$dsN<-as.numeric(tmj$ds)
tmj$bmsN<- as.numeric(tmj$bms)
tmj$jointN<-as.numeric(tmj$joint)
tmj$boneN<-as.numeric(tmj$bone)
```

2.2. Scale

```
tmj$ageS <- scale(tmj$age)
tmj$dsNS <- scale(tmj$dsN)
tmj$bmsNS <- scale(tmj$bmsN)
tmj$jointNS <- scale(tmj$jointN)
tmj$boneNS <- scale(tmj$boneN)
```

2.3. Partition of the data set into 80% training and 20% testing set

```
set.seed(180801)
ind <-sample(1:nrow(tmj),nrow(tmj)*0.8)
train <-tmj[ind,]
test <-tmj[-ind,]
```

3. Random forest (RF)

3.1. Load package “randomForest”

```
require(randomForest)

## Loading required package: randomForest

## randomForest 4.6-14

## Type rfNews() to see new features/changes/bug fixes.
```

3.2. Build model & predict

```
tmj.rf<-randomForest(as.factor(Group) ~ age + ds + bms + joint + bone,
                     train, importance=TRUE)
tmj.rf.prob <- predict(tmj.rf, type = "prob", newdata=test)
```

3.3. Looking inside the RF model

```
tmj.rf

##
## Call:
```

```
##  randomForest(formula = as.factor(Group) ~ age + ds + bms + joint +
bone, data = train, importance = TRUE)
##                Type of random forest: classification
##                      Number of trees: 500
## No. of variables tried at each split: 2
##
##         OOB estimate of  error rate: 19.67%
## Confusion matrix:
##     0  1 class.error
## 0 113 22   0.1629630
## 1  25 79   0.2403846
```

- this model has 500 trees

3.4. Importance of variables

```
importance(tmj.rf)

##              0         1 MeanDecreaseAccuracy MeanDecreaseGini
## age   18.65502 13.875118            21.638490         28.69686
## ds    31.44433 19.952991            33.977186         27.01476
## bms   15.91349 -3.670312             9.518296          5.29825
## joint 20.00317  8.646952            19.718832         10.92813
## bone  25.99064  5.026209            21.722192         11.70334
```

3.5. Importance plot

```
#png(filename = "importance.png", width =2000, height=2000, units="px", res
=300)
varImpPlot(tmj.rf, type=1, cex=2.0, pch=16, pt.cex=2, main="Variable import
ance plot of Random forest model")
```

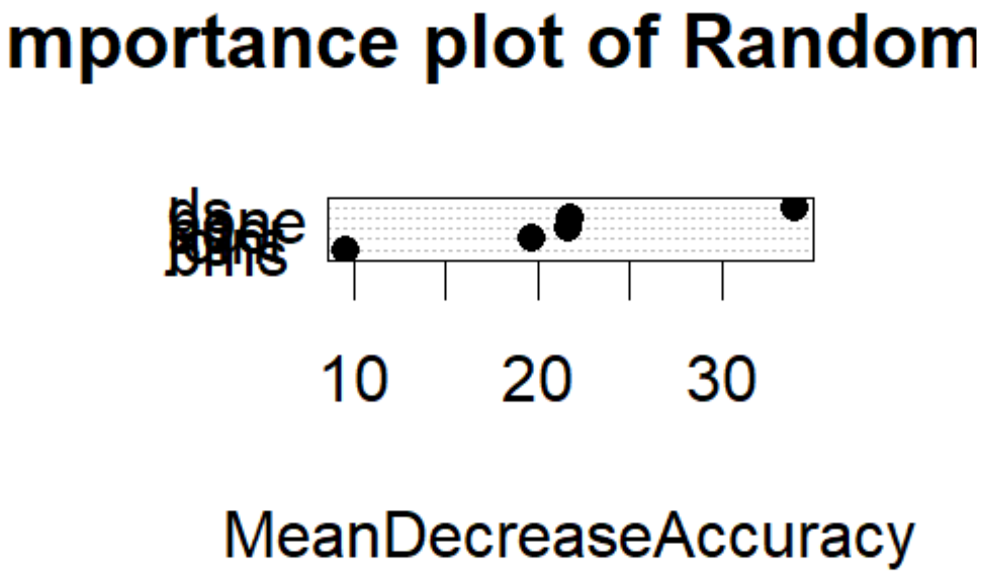

- type1= mean decrease in accuracy
- type2= mean decrease in node impurity

3.6. Export into .eps format

```
setEPS()
postscript("var importance plot.eps")
varImpPlot(tmj.rf, type=1, cex=2.0, pch=16, pt.cex=2, main="Variable import
ance plot of Random forest model")
```

4. Deep learning with keras

4.1. Loading libraries

```
#devtools::install_github("rstudio/tensorflow")
#install_tensorflow()
library(tensorflow)
library(keras)

## Warning: package 'keras' was built under R version 3.5.1

use_condaenv("tf-keras")
use_session_with_seed(617)

## Set session seed to 617 (disabled GPU, CPU parallelism)

library(yardstick)

## Loading required package: broom

## Warning: package 'broom' was built under R version 3.5.1

require(tidyquant)

## Loading required package: tidyquant

## Loading required package: lubridate

##
## Attaching package: 'lubridate'

## The following object is masked from 'package:base':
##
##     date

## Loading required package: PerformanceAnalytics

## Loading required package: xts

## Loading required package: zoo

##
## Attaching package: 'zoo'
```

```
## The following objects are masked from 'package:base':
##
##     as.Date, as.Date.numeric

##
## Attaching package: 'PerformanceAnalytics'

## The following object is masked from 'package:graphics':
##
##     legend

## Loading required package: quantmod

## Loading required package: TTR

## Version 0.4-0 included new data defaults. See ?getSymbols.

## Loading required package: tidyverse

## -- Attaching packages ------------------------------------------ tidyverse
 1.2.1 --

## v ggplot2 3.0.0     v purrr   0.2.4
## v tibble  1.4.2     v dplyr   0.7.6
## v tidyr   0.8.0     v stringr 1.3.0
## v readr   1.1.1     v forcats 0.3.0

## Warning: package 'ggplot2' was built under R version 3.5.1

## Warning: package 'dplyr' was built under R version 3.5.1

## -- Conflicts --------------------------------------------- tidyverse_conf
licts() --
## x lubridate::as.difftime() masks base::as.difftime()
## x dplyr::combine()         masks randomForest::combine()
## x lubridate::date()        masks base::date()
## x dplyr::filter()          masks stats::filter()
## x dplyr::first()           masks xts::first()
## x lubridate::intersect()   masks base::intersect()
## x dplyr::lag()             masks stats::lag()
## x dplyr::last()            masks xts::last()
## x ggplot2::margin()        masks randomForest::margin()
## x lubridate::setdiff()     masks base::setdiff()
## x readr::spec()            masks yardstick::spec()
## x lubridate::union()       masks base::union()

library(caret)

## Loading required package: lattice

##
## Attaching package: 'caret'

## The following object is masked from 'package:purrr':
##
##     lift
```

```
## The following objects are masked from 'package:yardstick':
##
##     mnLogLoss, precision, recall

## The following object is masked from 'package:tensorflow':
##
##     train

np<-import("numpy")
```

4.2. trainging and test sets for keras

```
keras_train <- subset(train, select=c(ageS, dsNS,bmsNS, jointNS, boneNS, Gr
oup))
keras_test <- subset(test, select=c(ageS, dsNS,bmsNS, jointNS, boneNS, Grou
p))
```

4.3. Predictor variable for the training and testing set

```
X_train <- keras_train[,-6]
X_test <- keras_test[,-6]
```

4.4. Target Variable for training and testing set

```
y_train <- keras_train[,6]
y_test <- keras_test[,6]
```

4.5. Multi-Layer Perceptron

```
model_keras <- keras_model_sequential()
model2 <- model_keras %>%
  layer_dense(units = 32, kernel_initializer = "uniform", activation = "rel
u",
              input_shape = ncol(X_train)) %>%
  layer_dropout(rate = 0.1) %>%

  layer_dense(units = 16, kernel_initializer = "uniform", activation = "rel
u") %>%
  layer_dropout(rate = 0.1) %>%
  layer_dense(units = 1, kernel_initializer = "uniform", activation = "sigm
oid")
```

4.6. Compile

```
compile2 <- model_keras %>%
  compile(optimizer = "rmsprop", loss = "binary_crossentropy", metrics = c
("accuracy"))

compile2

## Model
## ________________________________________________________________________
____
## Layer (type)                 Output Shape                 Param #
## ========================================================================
====
## dense_1 (Dense)               (None, 32)                   192
## ________________________________________________________________________
____
```

```
## dropout_1 (Dropout)              (None, 32)                  0
## ________________________________________________________________________
____
## dense_2 (Dense)                 (None, 16)                  528
## ________________________________________________________________________
____
## dropout_2 (Dropout)              (None, 16)                  0
## ________________________________________________________________________
____
## dense_3 (Dense)                 (None, 1)                   17
## ========================================================================
====
## Total params: 737
## Trainable params: 737
## Non-trainable params: 0
## ________________________________________________________________________
____
```

4.7. Fit the model to the training Data

```
kemodel <- fit(object = model2, x = as.matrix(X_train),
              y = y_train, batch_size = 100, epochs = 100,
              validation_split = 0.2, silent=TRUE)
```

4.8. Plotting the training / validation history of our Keras's Model

```
#png(filename = "learning.png", width =2400, height=3000, units="px", res=3
00)
plot(kemodel) + theme_tq(base_size=20) +
  scale_color_tq() +
  scale_fill_tq() +
  geom_point(size=2) +
  labs(title = "Deep Learning Training Result")
```

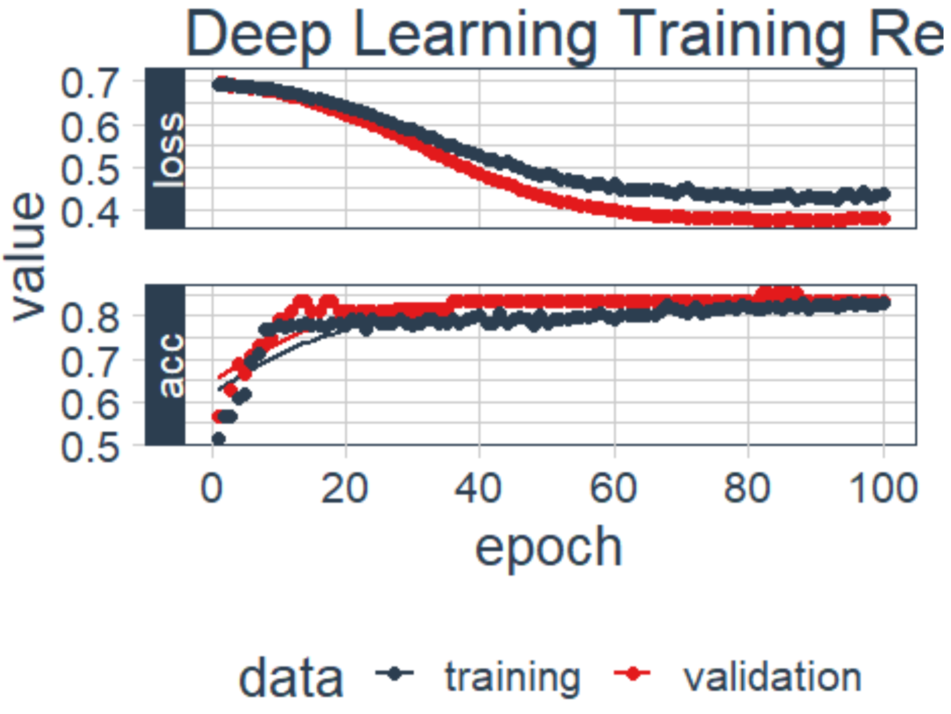

4.8.1. *Export into .eps format*

```
setEPS()
postscript("learning loss red and black.eps")
plot(kemodel) + theme_tq(base_size=20) +
  scale_color_tq() +
  scale_fill_tq() +
  geom_point(size=2) +
  labs(title = "Deep Learning Training Result")
```

4.9. Performance

4.9.1. *Predicted class*

```
pred_class <- predict_classes(object = model2,
                              x = as.matrix(X_test)) %>%  as.vector()
```

4.9.2. *Predicted class probability*

```
pred_prob <- predict_proba(object = model2,
                           x = as.matrix(X_test)) %>%  as.vector()
```

4.9.3. *Format test data and prediction*

```
predict_value <- tibble(truth = as.factor(y_test) %>% fct_recode(Yes = "1",
 No = "0"),
                        estimate = as.factor(pred_class) %>% fct_recode(Yes =
 "1", No = "0"),
                        pred_prob = pred_prob)

print(predict_value)

## # A tibble: 60 x 3
##    truth estimate pred_prob
```

```
##    <fct> <fct>       <dbl>
##  1 No    No          0.134
##  2 No    No          0.233
##  3 Yes   Yes         0.866
##  4 No    No          0.0583
##  5 Yes   Yes         0.854
##  6 No    No          0.340
##  7 Yes   No          0.242
##  8 Yes   Yes         0.591
##  9 Yes   Yes         0.505
## 10 No    No          0.105
## # ... with 50 more rows
```

4.9.4. Confusion table

```
predict_value %>% conf_mat(truth, estimate)

##           Truth
## Prediction No Yes
##        No  30   3
##        Yes  3  24
```

4.9.5. Accuracy

```
predict_value %>% metrics(truth, estimate)

## # A tibble: 1 x 1
##   accuracy
##      <dbl>
## 1      0.9
```

4.9.6. AUC of ROC

```
predict_value %>% roc_auc(truth,pred_prob)

## [1] 0.9399551
```

5. Plotting ROC with AUC of all Machine Learning models + single predictors

```
library(pROC)

## Type 'citation("pROC")' for a citation.

##
## Attaching package: 'pROC'

## The following objects are masked from 'package:stats':
##
##     cov, smooth, var

#setEPS()
#postscript("ROC of models.eps")
#png(filename = "ROC.png", width = 2000, height=2000, units="px", res=300)


# keras Neural Network
```

```r
roc.keras = roc(test[,'Group'], pred_prob, ci=TRUE)
plot(roc.keras, print.auc=TRUE, print.auc.cex=1.5, col='red', lwd=7,
     cex.axis=1.5, cex.lab=1.5, cex.main=2.0,
     print.auc.x= 0.58, print.auc.y = .16,
     main="ROC & AUC of models", legacy.axes=TRUE)

# RandomForest
roc.rf = roc(test[,'Group'],tmj.rf.prob[,2], ci=TRUE)
plot(roc.rf,  print.auc=TRUE, print.auc.cex=1.5, col='blue', lwd=7,
     print.auc.x= 0.58, print.auc.y = .09, add = TRUE, legacy.axes=TRUE)

# disc space alone
roc.ds=roc(form=train$Group~train$dsN, ci=TRUE)
plot(roc.ds, print.auc=TRUE, print.auc.cex=1.5, col='magenta1',lwd=7,
     print.auc.x= 0.58, print.auc.y = .020, add=TRUE, legacy.axes=TRUE)


legend(0.9, 0.22, c('Keras','RF', 'ds alone'),
       cex=1.6,x.intersp = 0.5, y.intersp = 1.0, bty = 'n',
       c('red', 'blue', 'magenta1'), xjust=0)
```

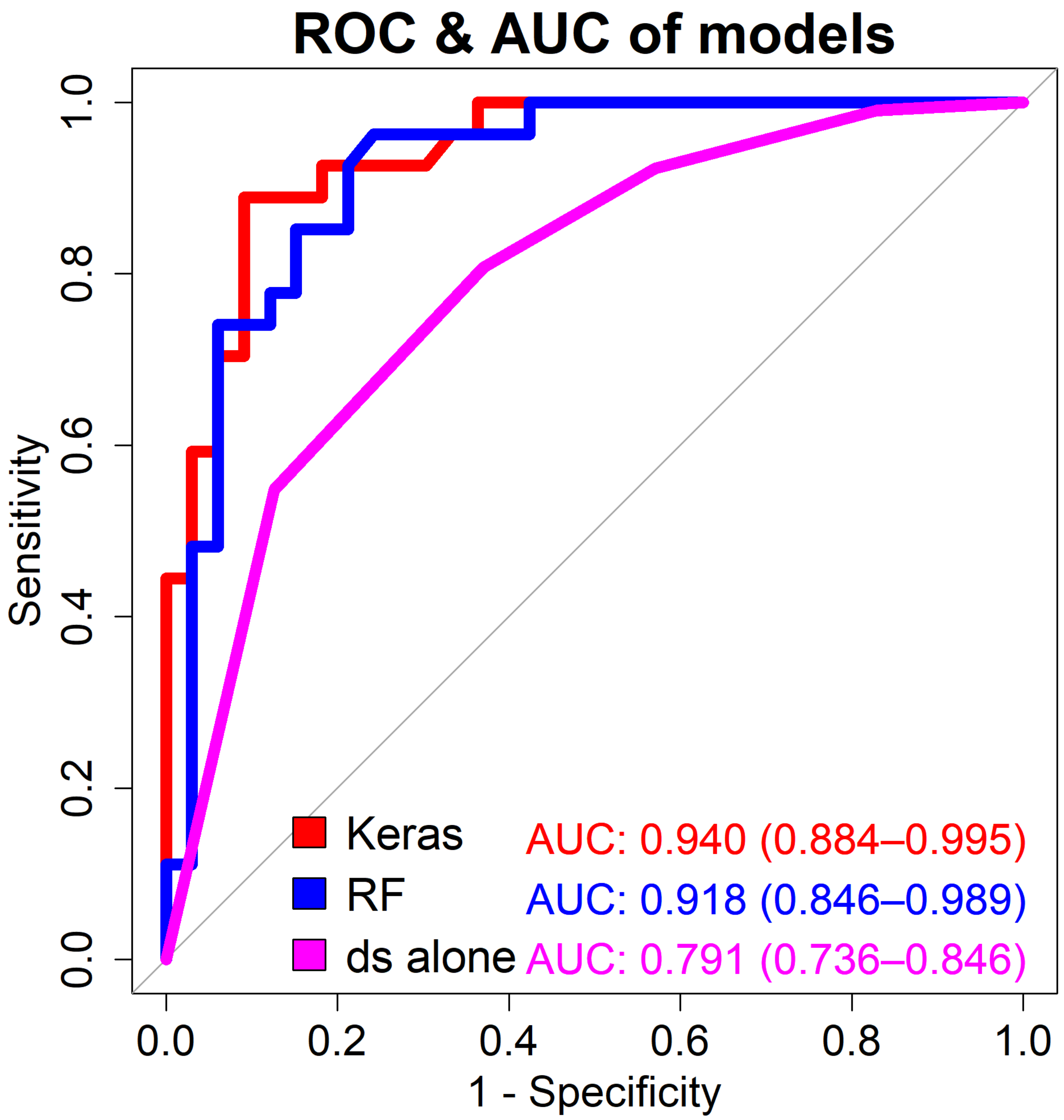

5.1. Testing if the differences between models are statistically significant

```
roc.test(roc.keras, roc.rf, alternative = "greater" )

## 
##  DeLong's test for two correlated ROC curves
## 
## data:  roc.keras and roc.rf
## Z = 0.92665, p-value = 0.1771
## alternative hypothesis: true difference in AUC is greater than 0
## sample estimates:
## AUC of roc1 AUC of roc2 
##   0.9399551   0.9175084

roc.test(roc.keras, roc.ds, alternative = "greater" )
```

```
##
##  DeLong's test for two ROC curves
##
## data:  roc.keras and roc.ds
## D = 3.7413, df = 187.12, p-value = 0.0001217
## alternative hypothesis: true difference in AUC is greater than 0
## sample estimates:
## AUC of roc1 AUC of roc2
##   0.9399551   0.7908832
```

```
roc.test(roc.rf, roc.ds, alternative = "greater" )
```

```
##
##  DeLong's test for two ROC curves
##
## data:  roc.rf and roc.ds
## D = 2.7399, df = 136.39, p-value = 0.003484
## alternative hypothesis: true difference in AUC is greater than 0
## sample estimates:
## AUC of roc1 AUC of roc2
##   0.9175084   0.7908832
```

6. Sensitivity, Specificity at Optimal cutpoints for each model

* Optimal cutpoint

- "youden": Youden's J statistic (Youden, 1950) is employed. The optimal cut-off is the threshold that maximizes the distance to the identity (diagonal) line. Can be shortened to "y". The optimality criterion is: max(sensitivities + specificities)

- "closest.topleft": The optimal threshold is the point closest to the top-left part of the plot with perfect sensitivity or specificity. Can be shortened to "c" or "t". The optimality criterion is: min((1 - sensitivities)^2 + (1- specificities)^2)

6.1. keras model

```
coords(roc.keras, x="best", input="threshold", best.method="youden")
```

```
##   threshold specificity sensitivity
##   0.4863707   0.9090909   0.8888889
```

```
coords(roc.keras, x="best", input="threshold", best.method="closest.toplef
t")
```

```
##   threshold specificity sensitivity
##   0.4863707   0.9090909   0.8888889
```

6.2. RF model

```
coords(roc.rf, x="best", input="threshold", best.method="youden")
```

```
##   threshold specificity sensitivity
##   0.1730000   0.7575758   0.9629630

coords(roc.rf, x="best", input="threshold", best.method="closest.topleft")

##   threshold specificity sensitivity
##   0.3960000   0.8484848   0.8518519
```

6.3. ds alone

```
coords(roc.ds, x="best", input="threshold", best.method="youden")

##   threshold specificity sensitivity
##   3.5000000   0.6296296   0.8076923

coords(roc.ds, x="best", input="threshold", best.method="closest.topleft")

##   threshold specificity sensitivity
##   3.5000000   0.6296296   0.8076923
```

7. Testing with imaginary data

```
imag.data<- read.csv("D:/tmj_keras_ex2.csv", sep=",", header=TRUE)
str(imag.data)

## 'data.frame':    1 obs. of  6 variables:
##  $ age  : int 65
##  $ ds   : int 3
##  $ bms  : int 2
##  $ joint: int 4
##  $ bone : int 1
##  $ perf : int 0
```

7.1. Scale imaginary data to the scale of previous dataset

```
imag.data$ageS <- scale(imag.data$age, attr(tmj$ageS, "scaled:center"), att
r(tmj$ageS, "scaled:scale"))
imag.data$dsNS <- scale(imag.data$ds, attr(tmj$dsNS, "scaled:center"), attr
(tmj$dsNS, "scaled:scale"))
imag.data$bmsNS <- scale(imag.data$bms, attr(tmj$bmsNS, "scaled:center"), a
ttr(tmj$bmsNS, "scaled:scale"))
imag.data$jointNS <- scale(imag.data$joint, attr(tmj$jointNS, "scaled:cent
er"), attr(tmj$jointNS, "scaled:scale"))
imag.data$boneNS <- scale(imag.data$bone, attr(tmj$boneNS, "scaled:center
"), attr(tmj$boneNS, "scaled:scale"))
imag.data<-subset(imag.data, select=c(ageS, dsNS, bmsNS, jointNS, boneNS, p
erf))
str(imag.data)

## 'data.frame':    1 obs. of  6 variables:
##  $ ageS   : num [1, 1] 2.87
##  $ dsNS   : num [1, 1] -0.342
##  $ bmsNS  : num [1, 1] 1.99
##  $ jointNS: num [1, 1] 2.73
##  $ boneNS : num [1, 1] -0.588
##  $ perf   : int 0
```

7.2. Predictor & Target variable for the imaginary data set

```
X_test <- imag.data[,-6]
y_test <- imag.data[,6]
```

7.3. Predicted class

```
pred_class_imag <- predict_classes(object = model2,
                                   x = as.matrix(X_test)) %>%  as.vector()
```

7.4. Predicted class probability

```
pred_prob_imag <- predict_proba(object = model2,
                                x = as.matrix(X_test)) %>%  as.vector()
```

7.5. format test data and prediction

```
predict_value_imag <- tibble(Dummy = as.factor(y_test) %>% fct_recode(Yes =
 "1", No = "0"),
                             estimate = as.factor(pred_class_imag) %>% fct_recode
(Yes = "1", No = "0"),
                             pred_prob = pred_prob_imag)

## Warning: Unknown levels in `f`: 1

## Warning: Unknown levels in `f`: 0

print(predict_value_imag)

## # A tibble: 1 x 3
##   Dummy estimate pred_prob
##   <fct> <fct>        <dbl>
## 1 No    Yes          0.939
```

–> high probability of having disc perforation
